# Effects of Astaxanthin on the Proliferation and Migration of Breast Cancer Cells In Vitro

**DOI:** 10.3390/antiox7100135

**Published:** 2018-10-04

**Authors:** Buckley McCall, Connor K. McPartland, Reece Moore, Anastasia Frank-Kamenetskii, Brian W. Booth

**Affiliations:** 1 Department of Biological Sciences, Clemson University, Clemson, SC 29634, USA; mccbuc@gmail.com (B.M.); reece5@g.clemson.edu (R.M.); 2 Department of Bioengineering, Clemson University, Clemson, SC 29634, USA; cmcpart@g.clemson.edu (C.K.M.); afrankk@g.clemson.edu (A.F.-K.)

**Keywords:** antioxidant, astaxanthin, breast cancer, carotenoid, migration, proliferation, Xanthoph

## Abstract

Astaxanthin (ASX) is a marine-based ketocarotenoid; an accessory pigment in plants in that it has many different potential functions. ASX is an antioxidant that is notably more potent than many other antioxidants. Antioxidants have anti-inflammatory and oxidative stress-reducing properties to potentially reduce the incidence of cancer or inhibit the expansion of tumor cells. In this study, we tested the hypothesis that ASX would inhibit proliferation and migration of breast cancer cells in vitro. We found that application of ASX significantly reduced proliferation rates and inhibited breast cancer cell migration compared to control normal breast epithelial cells. Based on these results, further investigation of the effects of ASX on not only breast cancer cells, but other forms of tumor cells, should be carried out.

## 1. Introduction

The accessory marine-based plant pigment astaxanthin (ASX) has many different potential functions [1]. ASX is a ketocarotenoid: carotenoids modulate cancer, reproduction, atherosclerosis, immunity, and age-related macular degeneration [2]. ASX is also a potent antioxidant, indicating that it has anti-inflammatory properties and oxidative stress-reducing properties [3]. Since ASX has anti-inflammatory and oxidative stress-reducing properties, in theory, the supplementation of ASX could positively affect cancer. Research shows that ASX maintains cell membrane structure while other carotenoids cause disorder in membranes [4]. ASX has proven to decrease to both oxidative stress and inflammation in a dose-dependent manner [5,6]. ASX has been able to inhibit inflammatory responses and oxidative stress via the activation of signaling pathways such as Nrf2-ARE in the brain [5]. ASX increases brain derived neurotropic factor (BDNF) protein levels, while concurrently decreasing oxidative stress levels [6]. ASX decreases the amount of inflammatory markers such as TNF-α, IL-6, and IFN-γ via NFκβ inhibition [7].

**Figure 1:**
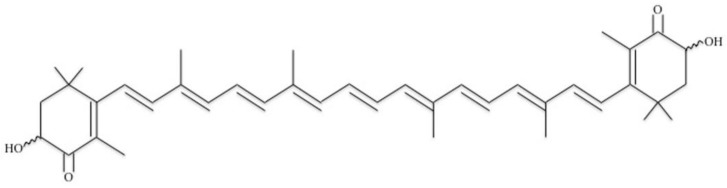
Chemical structure of the antioxidant astaxanthin.

ASX is a polar carotenoid that contains two oxygenated ends on each tail of the structure (Figure 1) [4]. The oxygenated ends are hydroxyl groups, which give the molecule minimal soluble properties. ASX is a long chain of carbon rings that have double bonds in every ring and double-bonded oxygen molecules on the end ring structures. The chemical formula for ASX is C_40_H_52_O_4_.

ASX may be beneficial in reducing cancer due to its inflammation and oxidative stress reducing properties. Its limited bioavailability may reduce the effectiveness of the supplement. ASX’s limited bioavailability decreases the ease of integration into the body. Using an emulsification-evaporation technique, ASX was incorporated into a delivery system, known as nanosystems, to address the bioavailability problem [8]. These nanosystems improve bioavailability by increasing the dissolution rate of ASX and the saturation solubility by reducing size and increasing surface area [8]. ASX has also been encapsulated for delivery via high-pressure homogenization and microchannel emulsification [9]. With this improved bioavailability problem, the ability to test ASX as a supplement in relation to cancer as well as other diseases is greatly enhanced.

ASX has been tested on different facets of cardiovascular disease and cancer, including oxidative stress and inflammation. ASX supplements prevent oxidative damage in smokers by activating antioxidants in the bodies of smokers [10]. ASX has greater effects than both Canthaxanthin and β-carotene when it comes to preventing and reducing oxidative stress in human dermal fibroblast cells incited with UVA-stimulation in order to induce oxidative stress [11].

ASX reduces oxidative stress, inflammation, and lipid levels in rats that received high fat diets [12] and has low toxicity and reduces mammary tumor size in rats that received *N*-methyl-*N*-nitroso-urea (MNU) to induce the mammary tumors [13]. ASX has a better and longer lasting effect when administered orally as a supplement in cats and dogs instead of being administered intravenously [14].

ASX has affected tumor growth in multiple different types of cancers. The antioxidant has notably resensitized pancreatic cells to the chemotherapy drug Gemcitabine [15]. The antioxidant had demonstrated anti-inflammatory properties in the ability to reduce prostate tumor growth as well as gastric inflammation [16,17]. The combination of ASX with vitamin C has been effective in the prevention and reduction of gastric inflammation [18,19]. ASX negatively affects breast cancer cell viability [20]. These inhibitory characteristics are related to the apoptotic and autophagic effects exhibited by the antioxidant. Targeted apoptosis and autophagy allow the antioxidant to kill the cancer cells without significantly affecting normal cells [21].

Breast cancer accounts for approximately one of every three cancers in women and is one of the most common diagnoses [22]. There are different types of breast cancer based on pathological examination: estrogen receptor (ER)^+^, human epidermal growth factor receptor 2 (HER2)^+^ and triple negative breast cancer (TNBC) (the absence of ER, HER2, and progesterone receptor (PR) expression).

In this set of experiments, we examined the effects on proliferation, migration, and gene expression on ER^+^ breast cancer cells and TNBC cells. We found significant differences induced by ASX in the cancer cells compared to normal breast epithelial cells.

## 2. Materials/Methods

### 2.1. Cell Lines

All cell lines were obtained from ATCC (Manassas, VA, USA). MCF7 and MDA-MB-231 cells were maintained in Dulbecco’s modified Eagle’s medium (DMEM) (Life Technologies, Grand Island, NY, USA) supplemented with 10% fetal bovine serum (FBS) (Atlanta Biologicals, Atlanta, GA, USA) and 1% antibiotic/antimycotic (Life Technologies). MCF10A cells were grown in DMEM supplemented with the MEGM bulletkit containing epidermal growth factor, insulin, bovine pituitary extract, and gentamicin (Lonza, Walkersville, MD, USA) with 10% fetal bovine serum and 1% antibiotic/antimycotic. All cells were grown at 37 °C with 5% CO_2_.

### 2.2. Astaxanthin

Valensa International (Eustis, FL, USA) provided 10% Astaxanthin (ASX) oleoresin. This oleoresin was dissolved in 10 mL aliquots of DMEM growth media in order to create concentrations of 0 μM, 10 μM, 25 μM, and 50 μM.

### 2.3. Migration Assays

Cell lines were each seeded into wells of 12-well plates (at 200,000 cells per well) and grown for 24 h. At that point, a 200-μL pipette tip was used to scratch a vertical line through the confluent cells at a perpendicular angle so that the central gap was consistently equidistant apart. After scratching the surface, an image was taken to serve as the before image. The cells were administered the dosage of media, media+dimethyl sulfoxide (DMSO; Life Technologies, Grand Island, NY, USA), 10 μM, 25 μM, or 50 μM of ASX. The cells were left to grow for 24 h; subsequently an after image was taken. Using the before and after images, the distance that the cells migrated into the gap was calculated and recorded. There were 30 distance measurements taken for each image pair in order to have a sufficient sample size and each experiment was repeated three times.

### 2.4. Proliferation Assays

The cells were grown and treated as described above. After 24 h the remaining cells were collected by trypsinization and counted using the hemocytometer. Each cell line had three replications.

### 2.5. RT-PCR

Following the aforementioned treatments, RNA was isolated using Trizol (Sigma, St. Louis, MO, USA) according to the manufacturer’s guidelines. Once RNA was isolated, cDNA was synthesized using a ThermoFischer Scientific cDNA synthesis kit (ThermoFisher, Waltham, MA, USA). RT-PCR was performed using BAX and BCL-2 oligo primers as well as GAPDH for a control (Table 1). The PCR products were separated on a 1.5% agarose gel, using ethidium bromide as the indicating agent. Gels were imaged using a FluorChemM (Cell Biosciences, Palo Alto, CA, USA). Band intensities were calculated using FluorChem software. BAX and BCL-2 intensities were normalized to GAPDH.

### 2.6. Statistical Analyses

Analyses were based on population means of the untreated cells with *z*-scores and *p*-values calculated. Standard deviations and standard error of the means were determined using Microsoft Excel. *p* values and *z* score were determined using https://www.socscistatistics.com/pvalues/normaldistribution.aspx and https://www.socscistatistics.com/tests/ztest/zscorecalculator.aspx, respectively. *p* values < 0.05 were considered significant.

## 3. Results

### ASX Inhibits Breast Cancer Cell Migration

**Figure 2:**
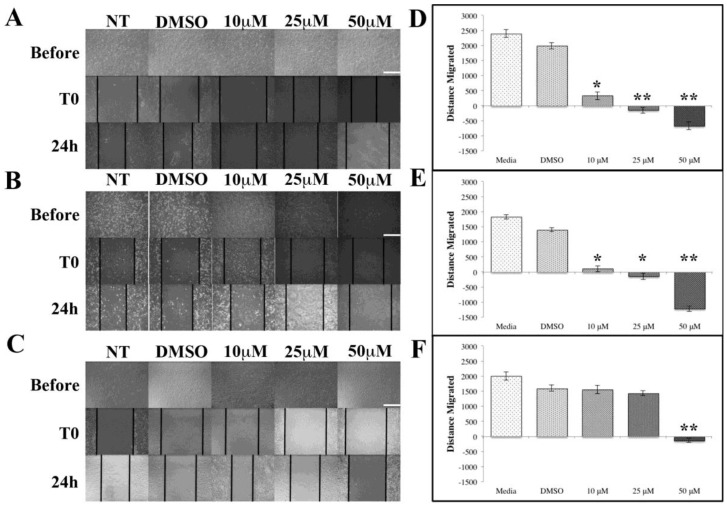
ASX inhibits cell migration. (**A**) Images of MCF7 cells prior to, immediately after, and 24 h post-scratch with indicated ASX concentrations; (**B**) MDA-MB-231 cells prior to, immediately after, and 24 h post-scratch with indicated ASX concentrations; (**C**) MCF10A cells prior to, immediately after, and 24 h post-scratch with indicated ASX concentrations. Scale bars = 50 μm; (**D**) Quantification of (**A**); (**E**) Quantification of (**B**); (**F**) Quantification of (**C**). * *p* < 0.05, ** *p* < 0.01.

Most cancer deaths are caused by metastases where tumor cells migrate. These three different cell lines encompass two breast cancer lines and one normal line to act as the experimental control. Figure 2 shows the migration assay results for each individual cell line. As shown in Figure 2A,B, the cells grow normally when cultured in media and DMSO settings. When the cells are exposed to ASX at increasing concentrations, the distance that the cells migrated declined until the cells started to recede at the 25 and 50 μM concentrations. MCF-7 ER^+^ breast cancer cells show an immediate decline in cell migration when ASX is administered. As the concentration increases from 10 μM to 25 μM and 50 μM, the results become significant to *p* < 0.01. Similar results were seen with the MDA-MB-231 TNBC cell line (Figure 2B). The two breast cancer cell lines both showed an immediate decrease in distance migrated as soon as ASX is administered. This decrease in distance traveled continued until the highest concentration (50 μM). The MCF-10A cell line is a control normal breast epithelial cell line. The distance migrated by the MCF10A cells did not decrease immediately with the addition of ASX, nor did it decrease when the concentration was increased to 25 μM (Figure 2C). The noticeable decrease in distance migrated only happened at the highest concentration of ASX suggesting 50 μM ASX is toxic to all cells used in these studies.

**Figure 3:**
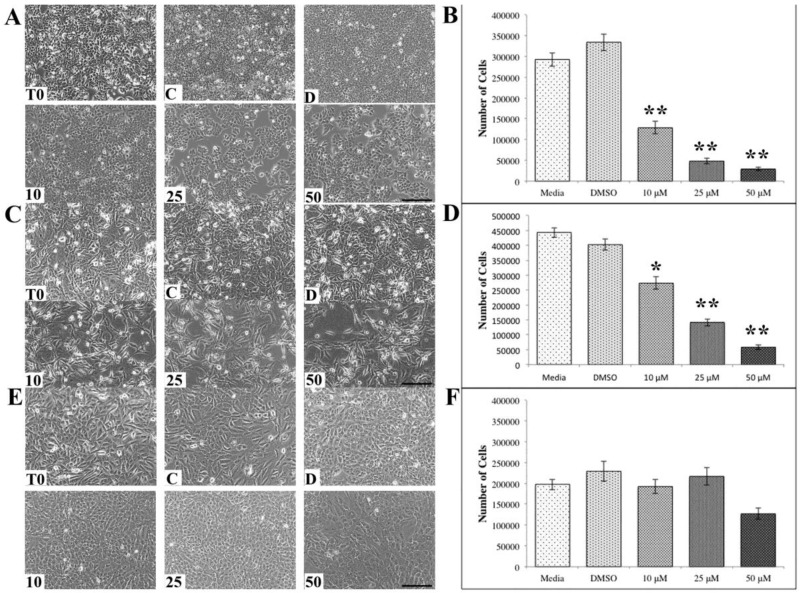
Astaxanthin (ASX) affects cell numbers. (**A**) MCF7 cells treated with ASX at concentrations indicated for 24 h; (**B**) quantification of (**A**); (**C**) MDA-MB-231 cells treated with ASX; (**D**) quantification of (**C**); and (**E**) MCF10A cells treated with ASX at concentrations indicated for 24 h and quantified in (**F**). (T0-time zero, C-untreated, D-DMSO, 10 μM, 25 μM, 50 μM). Scale bars = 100 μm. * *p* < 0.05, ** *p* < 0.01.

The observation that the higher concentrations of ASX was promoting an increase in gap distance in the migration studies displayed in Figure 2 suggests that the higher levels of ASX were killing the cells. To address this, cell counts were made following ASX treatments of 24 h for all three cell lines. After 24 h the number of ER+ MCF7 breast cancer cells was significantly reduced following ASX exposure at all concentrations (Figure 3A) as well as the numbers of the TNBC MDA-MB-231 breast cancer cells (Figure 3B). The reduction in cell numbers was concentration dependent. Conversely, the normal breast epithelial cells MCF10A showed no significant reduction in cell number following ASX exposure, even at the highest ASX concentration (Figure 3C). The number of MCF10A cells was reduced but not significantly.

**Figure 4:**
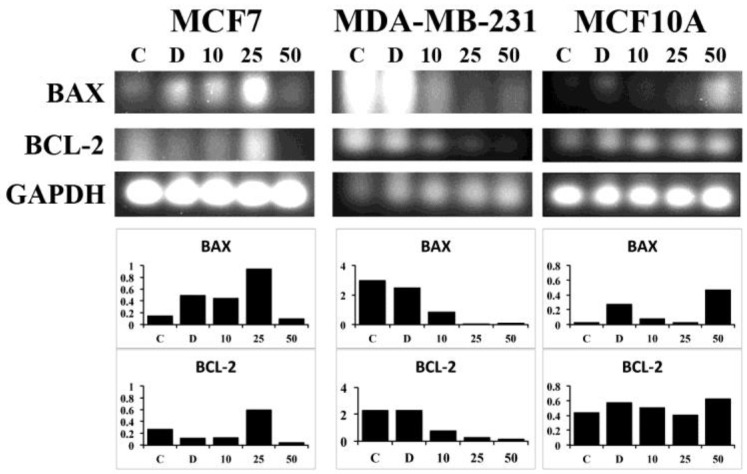
Gene expression of apoptotic mediators following ASX treatment. MCF7, MDA-MB-231, and MCF10A cells were treated with ASX for 24 h at the concentrations indicated. RT-PCR results are presented and the corresponding expression levels of the indicated gene normalized to GAPDH in arbitrary units. C-untreated, D-DMSO, 10 μM, 25 μM, and 50 μM.

Next, we wanted to determine if the reduction in cell number due to ASX exposure was due to apoptosis or another mechanism of cell death. Gene expression of two mediators of apoptosis, BAX and BCL-2, was investigated. In the MCF7 breast cancer cell line there was an increase in BAX, a proapoptotic marker, as the ASX concentration increased up to 25 μM (Figure 4, left). Expression of BCL-2 was highest after exposure to 25 μM ASX. Very little BAX or BCL-2 was found in cultures of MCF7 cells after exposure to the highest concentration of ASX, 50 μM. This may be due to reduced cell numbers due to increased ASX-induced cell death. In the triple-negative cell line MDA-MB-231 both BAX and BCL-2 mRNA levels were reduced following ASX treatments (Figure 4, middle). In the normal MCF10A cell line, the level of BCL-2 is consistent throughout the varying ASX concentrations (Figure 4, right), while BAX levels were elevated following treatment with 50 μM ASX.

## 4. Discussion

Research into the anticancer properties of natural compounds is a rapidly growing area of science. Many different classes of natural compounds have demonstrated anticancer properties including many plant-based and marine-based extracts [23,24,25,26,27]. ASX is a marine-based ketocarotenoid that has potent antioxidant characteristics [3]. Our data demonstrates that ASX significantly inhibits breast cancer cellular migration (Figure 2), significantly reduces breast cancer cell numbers (Figure 3), and induces apoptosis in breast cancer cells (Figure 4) compared to normal breast epithelial cells.

The goal of many cancer therapies, including radiation therapy and chemotherapy, is to halt the cellular division of the tumor cells. ASX blocks the proliferation and reduces cell numbers in the breast cancer cells investigated (Figure 3A,B). There are many side effects associated with both radiation therapy and chemotherapy [28,29,30]. Incorporation of ASX into anticancer therapy will help control tumor growth and potentially reduce the impact of radiation therapy and chemotherapy associated side effects.

Metastasis is the main cause of death in cancer patients [31]. Blocking metastasis would have a significant positive impact on cancer patient survival rates. One of the main cellular characteristics of metastasis is the migration of cancer cells from the initial tumors into the circulatory or lymphatic systems. Inhibiting this migration would reduce the number of metastases formed. ASX inhibits migration in the TNBC and ER^+^ breast cancer cells examined in these experiments. The MDA-MB-231 TNBC line is highly metastatic [32] but its migratory capacity is attenuated by ASX treatment.

No significant impacts on the migration, cell numbers, or change in apoptotic gene expression of the normal breast epithelial cell line MCF10A were observed following ASX treatments. The lack of effect of ASX on the normal breast cells is significant in that normal cells would still possess the capacity to migrate and proliferate in response to ASX cancer treatments meaning that the normal cells could continue to function normally and migrate into voids lefts by dying tumor cells in a healing process.

## 5. Conclusions

According to the collected data presented here, astaxanthin treatment is an effective method of reducing the proliferation and migration of breast cancer cells. Astaxanthin has shown demonstrated a consistent ability to reduce multiple types of cancer. These findings have the potential to incite many different types of medical research that could affect modern-day cancer treatment.

## Figures and Tables

**Table 1:** Primer sequences used for RT-PCR.

BAX Reverse primer (3′ antisense)	5′-CAT CTT CTT CCA GAT GGT GA-3′
BAX Forward primer (5′ sense)	5′-GTT TCA TCC AGG ATC GAG CAG-3′
BCL-2 Reverse primer (3′ antisense)	5′-GAG ACA GCC AGG AGA AAT CA-3′
BCL-2 Forward primer (5′ sense)	5′-CCT GTG GAT GAC TGA GTA CC-3′
GAPDH Reverse primer (3′ antisense)	5′-ACATCGCTCAGACCCATG-3′
GAPDH Forward primer (5′ sense)	5′-TGTAGTTGAGGTCAATGAAGGG-3′
